# Enhanced electrical properties of amorphous In-Sn-Zn oxides through heterostructuring with Bi_2_Se_3_ topological insulators

**DOI:** 10.1038/s41598-023-50809-7

**Published:** 2024-01-02

**Authors:** Chih-Chiang Wang, An-Ya Lo, Ming-Che Cheng, Yu-Sung Chang, Han-Chang Shih, Fuh-Sheng Shieu, Tzu-Hsien Tseng, He-Ting Tsai

**Affiliations:** 1 Department of Chemical and Materials Engineering, National Chin-Yi University of Technology, Taichung, 411030 Taiwan; 2 Department of Materials Science and Engineering, National Chung Hsing University, Taichung, 40227 Taiwan; 3 Department of Chemical and Materials Engineering, Chinese Culture University, Taipei, 11114 Taiwan; 4 Instrument Center, The Office of Research and Development, National Chung Hsing University, Taichung, 40227 Taiwan

**Keywords:** Materials science, Nanoscale materials

## Abstract

Amorphous indium tin zinc oxide (a-ITZO)/Bi_2_Se_3_ nanoplatelets (NPs) were fabricated using a two-step procedure. First, Bi_2_Se_3_ NPs were synthesized through thermal chemical vapor deposition at 600 °C on a glass substrate, and then a-ITZO was deposited on the surface of the Bi_2_Se_3_ NPs via magnetron sputtering at room-temperature. The crystal structures of the a-ITZO/Bi_2_Se_3_ NPs were determined via X-ray diffraction spectroscopy and high-resolution transmission electron microscopy. The elemental vibration modes and binding energies were measured using Raman spectroscopy and X-ray photoelectron spectroscopy. The morphologies were examined using field-emission scanning electron microscopy. The electrical properties of the a-ITZO/Bi_2_Se_3_ NPs were evaluated using Hall effect measurements. The bulk carrier concentration of a-ITZO was not affected by the heterostructure with Bi_2_Se_3_. In the case of the Bi_2_Se_3_ heterostructure, the carrier mobility and conductivity of a-ITZO were increased by 263.6% and 281.4%, respectively, whereas the resistivity of a-ITZO was reduced by 73.57%. This indicates that Bi_2_Se_3_ significantly improves the electrical properties of a-ITZO through its heterostructure, expanding its potential applications in electronic and thermoelectric devices.

## Introduction

Amorphous oxide semiconductors (AOSs) are attractive materials for applications in optoelectronics, thermoelectronics, and organic photovoltaics owing to their excellent properties, such as their high transparency, low deposition temperature, and high carrier mobility^1–3^. Several AOSs have been extensively studied, including zinc tin oxide (ZTO)^4^, indium zinc oxide (IZO)^5^, and indium gallium zinc oxide (a-IGZO)^6^. However, these AOSs have drawbacks, such as a high annealing temperature^7,8^, high off‒current^9^, and relatively low carrier mobility (~ 10 cm^2^/V-s)^10,11^.

In addition to the aforementioned AOS materials, amorphous indium tin zinc oxide (a-ITZO) has attracted considerable attention owing to its advantageous characteristics, including large carrier mobility and high carrier concentrations^12,13^. The primary factor contributing to the high electron carrier mobility within the conduction‒band minimum of a-ITZO is the increasing overlap area between the orbitals of In 5 s and Sn 5 s. These orbitals possess strong divergence, high symmetry, and an electronic configuration similar to that of (n‒1)d^10^n^0^ (n ≥ 4)^14–16^. In addition to the improved carrier mobility, the carrier concentration of a-ITZO is increased. This is achieved by substituting lattice In^3+^ ions with Zn^2+^ and Sn^4+^ ions, which form acceptor defects of ${\left({{{\text{Zn}}}^{2+}}_{{{\text{In}}}^{3+}}\right)}^{\mathrm{^{\prime}}}$ and donor defects of ${\left({{{\text{Sn}}}^{4+}}_{{{\text{In}}}^{3+}}\right)}^{\bullet }$. The presence of Zn^2+^ induces lattice distortion through the Jahn‒Teller effect, leading to the formation of oxygen vacancies (${\left({{{\text{V}}}^{0}}_{{{\text{O}}}^{2-}}\right)}^{\bullet \bullet }$) as donor defects. Consequently, the carrier concentration in a-ITZO increases^11^. Additionally, the carrier mobility of a-ITZO is approximately 20‒30 cm^2^/V-s^13,17–22^. Improvements in the electrical properties of a-ITZO are necessary for optimizing the electrical performance^16^. The focus should be on enhancing the carrier mobility rather than solely increasing the carrier concentration^11^. Dopants have been proven to be effective for enhancing the electrical properties of AOS materials. Several types of doped ITZO have been extensively investigated, including Mg-^17^, Al-^23^, Er-^24^, P-^1^, W-^25^, and Pr-doped ITZO^26^. Heterostructures combining transparent conducting oxides with metals provide another approach for improving the electrical performance. Examples include indium tin oxide/silver/indium tin oxide (ITO/Ag/ITO)^27^, aluminum-doped zinc oxide/silver/aluminum-doped zinc oxide (AZO/Ag/AZO)^28^, zinc-tin-oxide/silver/indium-tin-oxide (ZTO/Ag/ITO)^29^, gallium zinc oxide/silver/gallium zinc oxide (GZO/Ag/GZO)^30^, and indium zinc oxide/gold/indium zinc oxide (IZO/Au/IZO) heterostructures^31^.

Rhombohedral bismuth selenide (Bi_2_Se_3_) is a direct n-type topological insulator with a narrow band‒gap of 0.35 eV^32^. The bulk structure of Bi_2_Se_3_ consists of five stacked atomic layers, i.e., ${{\text{Se}}}_{1}-{{\text{Bi}}}_{1}-{{\text{Se}}}_{1}^{\mathrm{^{\prime}}}-{{\text{Bi}}}_{1}-{{\text{Se}}}_{1}$, and is referred to as a quintuple layer (QL)^33^. Within the QLs, covalent bonds between Se and Bi dominate, whereas van der Waals (vdW) forces govern the bonding between QLs^34^. Bi_2_Se_3_ is a unique material because of its insulating bulk state and gapless conducting surface state, which are attributed to the spin‒orbital coupling (SOC) and time‒reversal symmetry (TRS)^35,36^. These properties prevent the surface backscattering effect caused by non-magnetic impurities, resulting in efficient electron transport at the surface^37,38^. Bi_2_Se_3_ exhibits a high electron carrier mobility of up to 600 cm^2^ V^−1^ s^−1^^39^. Consequently, Bi_2_Se_3_, with its gapless conducting surface state, has several notable features, including (a) photon-like and spin-polarized electrons, (b) a low power dissipation rate, and (c) the quantum spin Hall effect^34,39–41^.

In this study, Bi_2_Se_3_ NPs were fabricated on a glass substrate via thermal chemical vapor deposition (CVD), followed by the deposition of a-ITZO via magnetron sputtering. Subsequently, the electrical properties of the ITZO/Bi_2_Se_3_ NPs, including the bulk carrier concentration, carrier mobility, resistivity, and conductivity were analyzed via Hall effect measurements at room-temperature.

## Results and discussions

### Crystal structures

**Figure 1:**
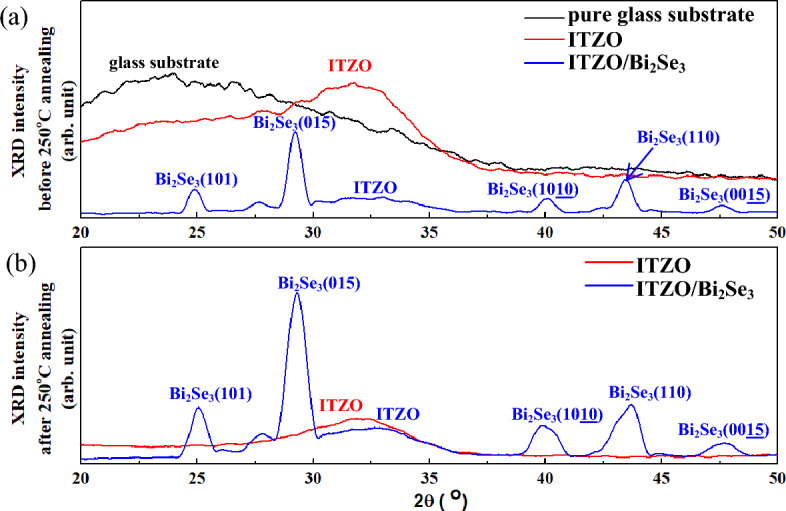
XRD patterns of pure glass, ITZO thin film, and ITZO/Bi_2_Se_3_ NPs (**a**) before and (**b**) after annealing at 250 °C.

Figure 1a,b show the XRD patterns of the glass substrate, ITZO thin films, and Bi_2_Se_3_ and ITZO/Bi_2_Se_3_ NPs before and after annealing at 250 °C. As shown in Fig. 1a, the glass substrate exhibited a broad peak at approximately 24.5°^42^, whereas the ITZO thin film exhibited a broad hump centered at approximately 31.58°, indicating an amorphous structure^43^. The d_ITZO_-spacing at 2θ = 31.58° was approximately 0.285 nm estimated using Bragg’s law^44,45^. As shown in Fig. 1a, the ITZO/Bi_2_Se_3_ NPs exhibited six significant peaks at 2θ = 24.88°, 29.21°, 32.81°, 40.06°, 43.42°, and 47.56°, which corresponded to the Bi_2_Se_3_(101), Bi_2_Se_3_(015), ITZO, Bi_2_Se_3_(1010), Bi_2_Se_3_(110), and Bi_2_Se_3_(0015) planes^32,43^, respectively. After the annealing treatment at 250 °C, as shown in Fig. 1b, the ITZO thin film and ITZO/Bi_2_Se_3_ NPs exhibited broad peaks at approximately 31.87° and 32.7°, respectively, indicating an amorphous ITZO phase. The six significant peaks in Fig. 1b are similar to those in Fig. 1a, and correspond to the relevant Bi_2_Se_3_ and ITZO crystal planes. These results confirm that the Bi_2_Se_3_ phase was stable under the annealing at 250 °C.

### Fine structures

**Figure 2:**
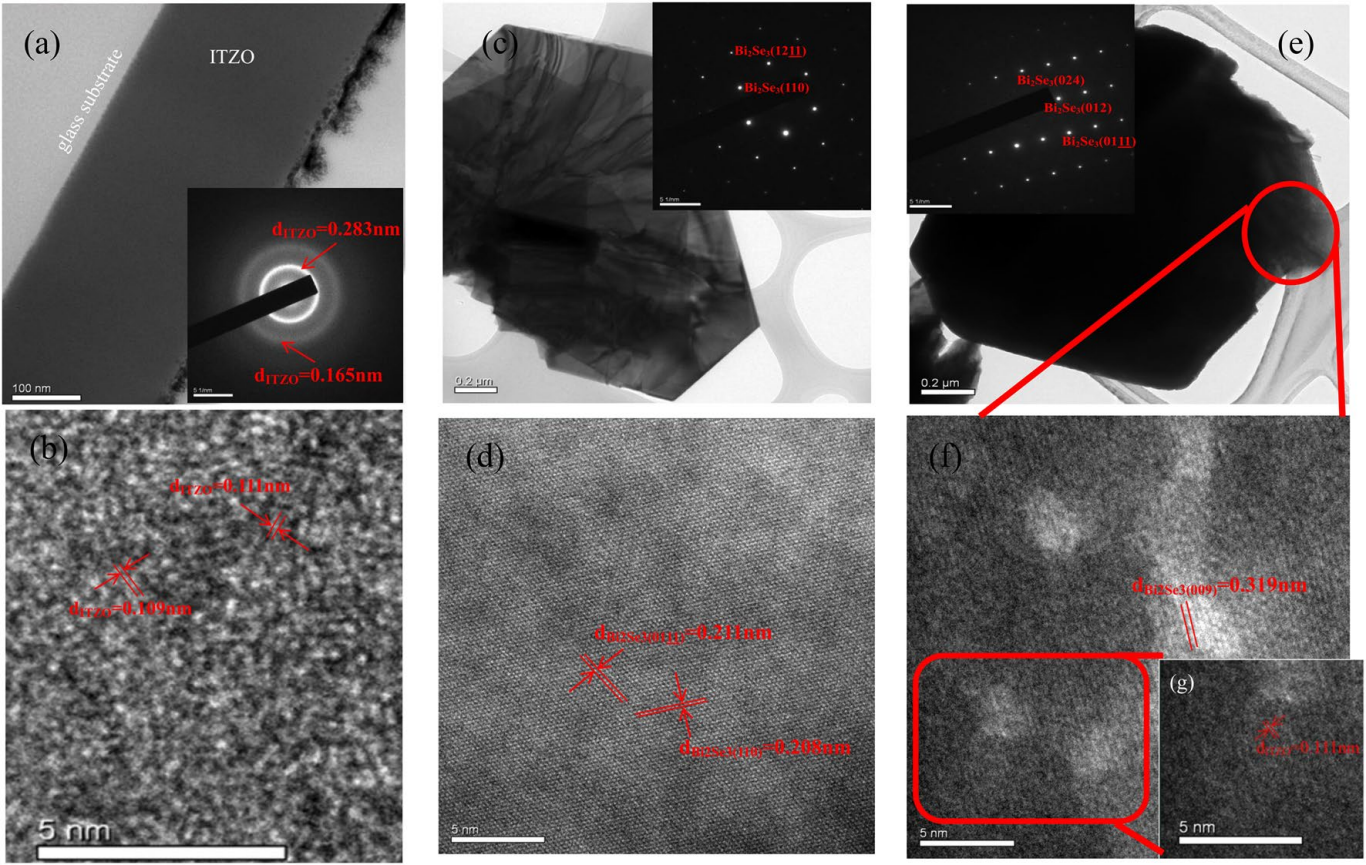
Low-magnitude and HRTEM iamges of (**a**), (**b**) ITZO thin film, (**c**,**d**) Bi_2_Se_3_ NPs, and (**e**,**f**) ITZO/Bi_2_Se_3_ NPs, respectively. The insets are the HRTEM-SAD patterns. (**g**) the zoomed-in image of (f).

Figure 2a shows a low-magnitude TEM image of a 270‒nm‒thick ITZO thin film deposited on a pure glass substrate after annealing at 250 °C. The inset presents the HRTEM-selected-area electron diffraction (SAED) pattern of ITZO, indicating an amorphous structure. The SAED pattern exhibits two significant rings with inner and outer d-spacings of 0.283 and 0.165 nm, respectively. The ITZO used in this study was composed of 85 wt% In_2_O_3_, 10 wt% SnO_2_, and 5 wt% ZnO, indicating that the In_2_O_3_ was the host material. Sn^4+^ and Zn^2+^ ions prefer to replace In^3+^ ions at the b- and d-sites in the In_2_O_3_ lattice^11^. Hence, the d_ITZO_-spacings of 0.283 and 0.165 nm were related to the In_2_O_3_(321) and In_2_O_3_(611) planes (JCPDS 71-2195), respectively. The former result is agreed with the XRD results as shown in Fig. 1. Figure 2b shows an HRTEM image of ITZO indicating a disordered lattice with estimated d_ITZO_-spacings of 0.111 and 0.109 nm, which correspond to the In_2_O_3_(833) and In_2_O_3_(248) planes, respectively, according to JCPDS 71–2195. The HRTEM-energy-dispersive X-ray spectroscopy (EDS) spectrum shown in Fig. S1a confirms the presence of In, Sn, and Zn. Figure 2c presents a low-magnitude TEM image of hexagonal-shaped Bi_2_Se_3_ NPs after annealing at 250°C. The HRTEM-SAED pattern shown in Fig. 2c exhibits peaks corresponding to the Bi_2_Se_3_(1211) and Bi_2_Se_3_(110) planes. The HRTEM image of the Bi_2_Se_3_ NPs in Fig. 2d shows d-spacings of 0.211 and 0.208 nm, corresponding to the Bi_2_Se_3_(0111) and Bi_2_Se_3_(110) planes, respectively. Figure 2e shows a low-magnitude TEM image of the ITZO/Bi_2_Se_3_ NPs after annealing at 250°C. The HRTEM-SAED pattern shown in the inset of Fig. 2e exhibits peaks corresponding to the Bi_2_Se_3_ planes of (024), (012), and (0111). The HRTEM image presented in Fig. 2f shows a d-spacing of 0.319 nm, corresponding to the Bi_2_Se_3_(009) plane. In the zoomed-in HRTEM image shown in Fig. 2g, the d-spacing of d_ITZO_ was estimated to be 0.111 nm, corresponding to the In_2_O_3_(833) plane. The presence In, Sn, Zn, Bi, and Se was confirmed using HRTEM-EDS, as shown in Fig. S1b. These results indicated that the ITZO was deposited on the surface of the Bi_2_Se_3_ NPs, and formed ITZO/Bi_2_Se_3_ heterostructures.

### Surface morphologies

**Figure 3:**
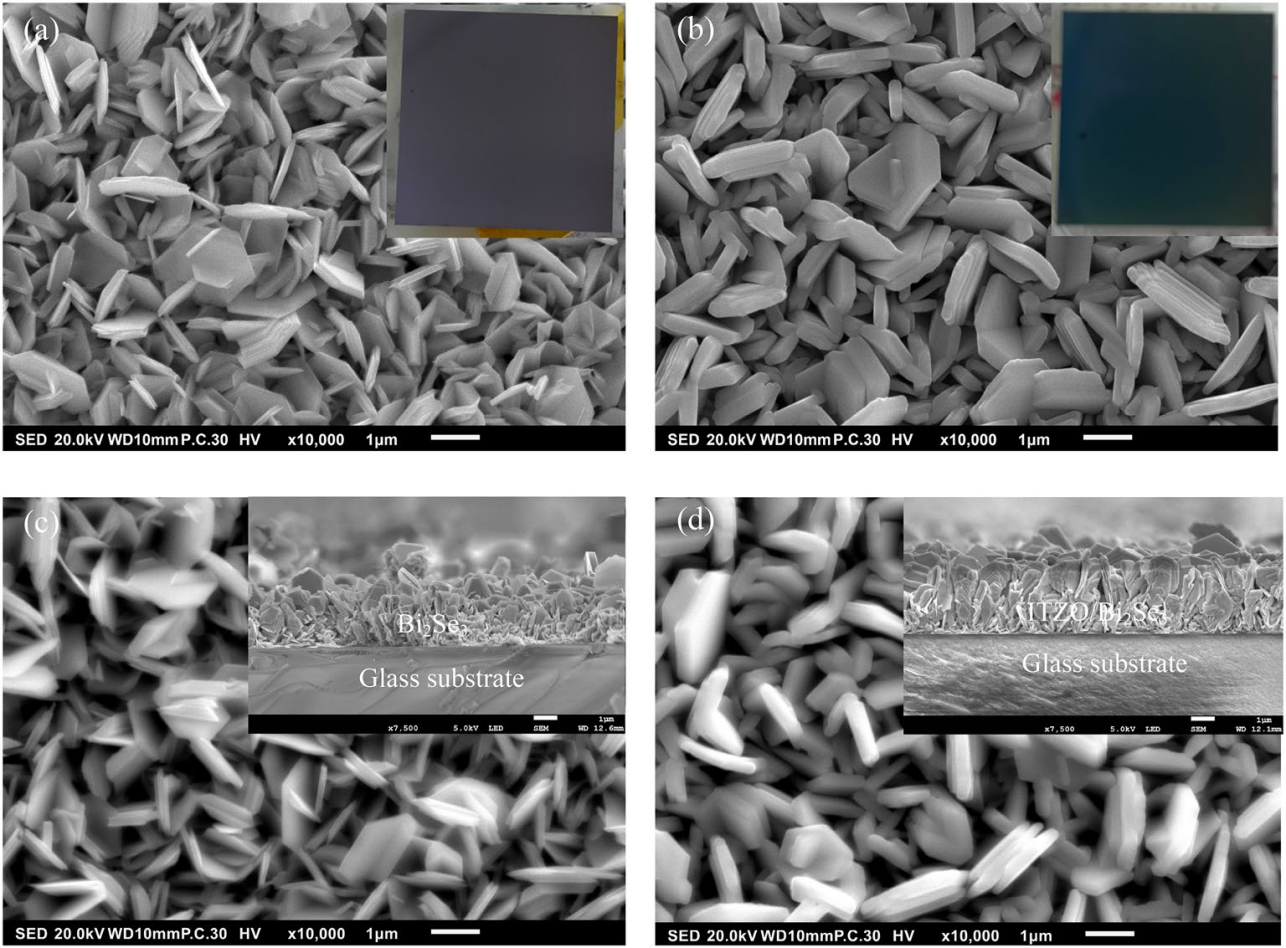
FESEM images of the Bi_2_Se_3_ and ITZO/Bi_2_Se_3_ NPs of (**a**,**b**) before, and (**c**,**d**) after annealing at 250 °C, respectively. The insets in (**a**,**b**) show the photographs of the Bi_2_Se_3_ and ITZO/Bi_2_Se_3_ NPs before annealing at 250 °C, respectively. The insets in (**c**,**d**) are the cross-section images of Bi_2_Se_3_ and ITZO/Bi_2_Se_3_ NPs after annealing at 250 °C.

Figure 3a–d show FESEM images of Bi_2_Se_3_ and ITZO/Bi_2_Se_3_ NPs before and after annealing at 250 °C. They exhibit a hexagonal shape, as observed in the HRTEM images of Fig. 2c,d. The average thicknesses of these NPs were estimated using the ImageJ software, and the results indicated that each Bi_2_Se_3_ NPs had a thicknesses of approximately 71.8 nm (divided by 20 pieces) and 86.8 nm (divided by 20 pieces) before and after annealing at 250 °C, respectively. The thickness of each QL was approximately 0.955 nm^46^. Thus, the average number of QLs was 83 for each pristine Bi_2_Se_3_ NPs. On average, each ITZO/Bi_2_Se_3_ NPs had thicknesses of 247.3 and 232.5 nm before and after annealing at 250 °C, respectively. These results explain why the ITZO/Bi_2_Se_3_ NPs shown in Fig. 2e is not transparent. The thickness of the covered ITZO thin films was estimated to be approximately 160 nm. Photographs of the Bi_2_Se_3_ and ITZO/Bi_2_Se_3_ NPs before annealing at 250 °C are presented in the insets of Fig. 3a,b. Their colors differed significantly; the Bi_2_Se_3_ NPs were grey, whereas the ITZO/Bi_2_Se_3_ NPs were yellowish-green. The cross-sectional SEM images in the insets of Fig. 3c,d indicate the total deposition thicknesses of the Bi_2_Se_3_ and ITZO/Bi_2_Se_3_ NPs after annealing at 250 °C, which were approximately 2.2 and 2.5 μm, respectively. The SEM–EDS results for the Bi_2_Se_3_ and ITZO/Bi_2_Se_3_ NPs are presented in Fig. S2a,b, which confirm the existence of Bi, Se, In, Sn, and Zn.

### Vibration modes

**Figure 4:**
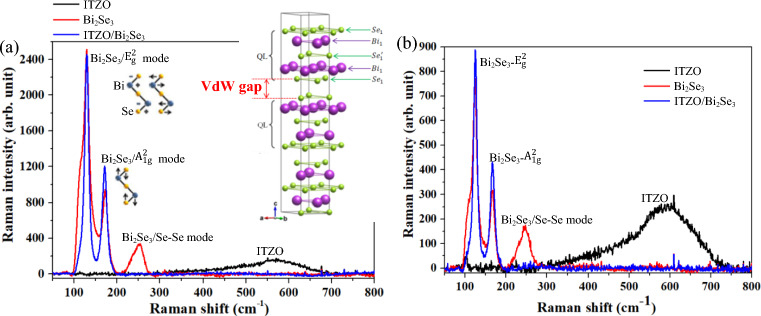
Raman spectra of ITZO thin film, and Bi_2_Se_3_ and ITZO/Bi_2_Se_3_ NPs (**a**) before and (**b**) after annealing at 250 °C. The inset shows the typical layered structure of Bi_2_Se_3_^47^.

Figure 4a,b show the Raman spectra of the ITZO thin film and Bi_2_Se_3_ and ITZO/Bi_2_Se_3_ NPs before and after annealing at 250 °C. The Raman spectrum of ITZO before and after annealing at 250 °C exhibited a broad peak in the range of 300‒700 cm^−1^, which was centered at approximately 563.01 and 587.78 cm^−1^, respectively. The Bi_2_Se_3_ NPs exhibited three significant vibration modes of ${{\text{E}}}_{{\text{g}}}^{2}$, ${{\text{A}}}_{1{\text{g}}}^{2}$, and Se-Se bonds^47,48^ before annealing at 250 °C at wavenumbers of 126.69, 170.51, and 248.49 cm^−1^, respectively, as shown in Fig. S3a. In addition, the ITZO/Bi_2_Se_3_ NPs exhibited two significant modes of ${{\text{E}}}_{{\text{g}}}^{2}$ and ${{\text{A}}}_{1{\text{g}}}^{2}$ at 128.99 and 170.64 cm^−1^ before annealing at 250 °C, as shown in Fig. S3b, while the Se–Se mode was suppressed. After annealing at 250 °C, the Bi_2_Se_3_ NPs exhibited the same vibration modes (${{\text{E}}}_{{\text{g}}}^{2}$, ${{\text{A}}}_{1{\text{g}}}^{2}$, and Se-Se at 122.37, 165.90, and 244.67 cm^−1^, respectively, as shown in Fig. S3c) as before the annealing at 250 °C, as did the ITZO/Bi_2_Se_3_ NPs (${{\text{E}}}_{{\text{g}}}^{2}$ and ${{\text{A}}}_{1{\text{g}}}^{2}$ at 124.66 and 166.67 cm^−1^, respectively, as shown in Fig. S3d).

Bi_2_Se_3_ has a layered crystal structure, as shown in the inset of Fig. 4a^49^, where each layer comprises five monoatomic layers, i.e., ${{\text{Se}}}_{1}-{{\text{Bi}}}_{1}-{{\text{Se}}}_{1}^{\mathrm{^{\prime}}}-{{\text{Bi}}}_{1}-{{\text{Se}}}_{1}$; therefore, it is called a quintuple layer (QL). Covalent bonds dominate the bonding within the QL, and the vdW force connects the QLs^32^. ${{\text{E}}}_{{\text{g}}}^{2}$ is a Raman active mode, i.e., the in-plane symmetric bending mode associated with the shearing of the upper/lower ${{\text{Se}}}_{1}-{{\text{Bi}}}_{1}$ bond in the opposite vibration direction. ${{\text{A}}}_{1{\text{g}}}^{2}$ is a Raman active mode similar to ${{\text{E}}}_{{\text{g}}}^{2}$ and represents the out-of-plane symmetric stretching of the upper/lower ${{\text{Se}}}_{1}-{{\text{Bi}}}_{1}$ bond in the opposite vibration direction^34,47^. The Se–Se vibration mode is assigned to the in-plane vibration of the topmost hexagonal network of Se atoms in Bi_2_Se_3_ layered structures^48,50^. Therefore, the Se-Se vibration mode was observed in the Bi_2_Se_3_ NPs at 248.49 and 244.67 cm^−1^ before and after annealing at 250 °C, respectively. The Se–Se vibration mode of the Bi_2_Se_3_ NPs was suppressed after the NPs were covered with the ITZO thin film, as shown in Fig. 4a (before annealing) and b (after annealing), implying that the topmost ${{\text{Se}}}_{1}$ atoms in the Bi_2_Se_3_ layered structure bonded with the ITZO thin film, suppressing the in-plane Se-Se vibration. These results confirmed that ITZO/Bi_2_Se_3_ NPs were successfully fabricated.

### Binding energies

**Figure 5:**
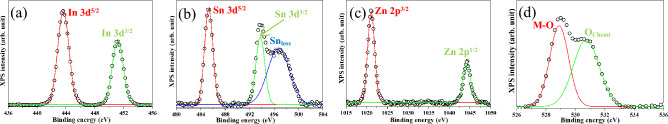
XPS spectra of ITZO thin film of (**a**) In 3d, (**b**) Sn 3d, (**c**) Zn 2p, and (**d**) O 1 s before annealing at 250 °C.

Figure 5a–d show the X-ray photoelectron spectra (XPS) of the ITZO thin film for the In 3d, Sn 3d, Zn 2p, and O 1 s orbitals, respectively. In Fig. 5a, the spectrum is split into two peaks of In 3d^5/2^ and In 3d^3/2^ at 443.63 and 451.18 eV with an energy difference of 7.55 eV, indicating that In mainly existed in a trivalent form (In^3+^) in the In_2_O_3_ lattice^51^. In Fig. 5b, the spectrum for the Sn 3d orbital is split to 485.29 and 493.74 eV peaks, which correspond to Sn 3d^5/2^ and Sn 3d^3/2^, respectively, indicating the presence of tetravalent Sn (Sn^4+^) in the SnO_2_ lattice^52^. The broad peak centered at 496.72 eV near Sn 3d^3/2^ is related to the Sn-loss signal (Sn_loss_)^52^, implying that the ITZO thin film had a high conductivity^53^. In Fig. 5c, the splitting of spin‒orbit doublets of Zn 2p^3/2^ and Zn 2p^1/2^ is observed at energy of 1021.08 and 1044.25 eV, which is assigned to the divalent zinc (Zn^2+^) in the ZnO lattice^54^. In Fig. 5d, the O1s orbital is deconvoluted into two peaks at approximately 528.89 and 530.73 eV. The former is related to the oxygen bonded with the metal forming the metal–oxygen (M–O) bonds in the metallic oxides, whereas the latter is attributed to the chemisorbed oxygen (O_chemi_: O_2_^−^, O^−^ etc.) on the nanostructure surface^52,54,55^. These results confirmed that Sn^4+^ and Zn^2+^ replaced In^3+^ in the lattice.

**Figure 6:**
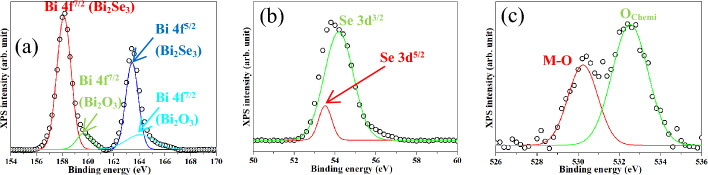
XPS spectra of Bi_2_Se_3_ NPs of (**a**) Bi 4f, (**b**) Se 3d, and (**c**) O 1 s.

Figure 6a–c show the XPS spectra of the Bi_2_Se_3_ NPs. In Fig. 6a, four peaks are observed at 158.12, 163.43, 159.68, and 164.06 eV, respectively. The first two peaks are attributed to the Bi 4f^7/2^ and Bi 4f^5/2^ orbitals of Bi^3+^ in the Bi_2_Se_3_ lattice, whereas the last two peaks are assigned to the Bi 4f^7/2^ and Bi 4f^5/2^ orbitals of Bi^3+^ in the Bi_2_O_3_ lattice^34^. In Fig. 6b, a broad peak is deconvoluted into peaks at 53.51 and 54.18 eV, respectively. The former peak is related to the divalent Se (Se^2-^) of Se 3d^5/2^ in the Bi_2_Se_3_ lattice, whereas the latter is ascribed to Se 3d^3/2^^56^. In Fig. 6c, O 1 s peaks are observed at 530.23 and 532.52 eV. The former peak is related to the oxygen binding energy of the metal‒oxygen bonds in the metal‒oxide lattice, and the latter peak corresponds to the chemisorbed oxygen on the nanostructure surface.

**Figure 7:**
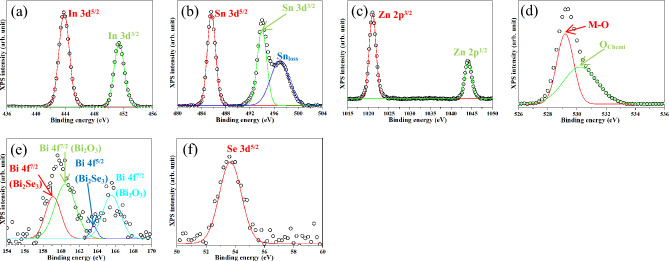
XPS spectra of ITZO/Bi_2_Se_3_ NPs of (**a**) In 3d, (**b**) Sn 3d, (**c**) Zn 2p, (**d**) O 1 s, (**e**) Bi 4f, and (**f**) Se 3d.

Figure 7 presents the In 3d (Fig. 7a), Sn 3d (Fig. 7b), Zn 2p (Fig. 7c), O 1 s (Fig. 7d), Bi 4f. (Fig. 7e), and Se 3d (Fig. 7f) XPS spectra of the ITZO/Bi_2_Se_3_ NPs. As shown in Fig. 7a, the In 3d spectrum is split into two peaks at approximately 443.85 and 451.41 eV, implying the splitting of In^3+^ into the In 3d^5/2^ and In 3d^3/2^ orbitals in the In_2_O_3_ phase. The Sn 3d orbital presented in Fig. 7b shows three peaks at 485.62, 493.99, and 496.91 eV. The first two peaks are attributed to Sn 3d^5/2^ and Sn 3d^3/2^, indicating the presence of Sn^3+^ in the SnO_2_ lattice. The last peak is assigned to Sn_loss_, which is easily detected in highly conductive materials such as ITZO. The Zn 2p peak is observed two peaks located at 1021.04 and 1044.13 eV, as shown in Fig. 7c. The former peak was ascribed to Zn 2p^3/2^, and the latter peak corresponded to Zn 2p^1/2^, implying the presence of Zn^2+^ in the ZnO lattice. The O 1 s peak was deconvoluted into two peaks at 529.20 and 530.22 eV, which were related to the metal‒oxygen bonds in the metal-oxide lattices and the chemisorbed oxygen on the nanostructure surface, respectively, as shown in Fig. 7d. The signal intensities of Bi 4f. and Se 3d were reduced owing to the ITZO covering, as shown in Fig. 7e,f. Relevant Bi_2_Se_3_ lattice peaks of Bi 4f^7/2^ (159.11 eV), Bi 4f^5/2^ (163.58 eV), and Se 3d^5/2^ (53.67 eV) were still detected. In addition, the related Bi_2_O_3_ peaks of Bi 4f^7/2^ at 160.45 eV and Bi 4f^5/2^ at 165.56 eV were observed, even when the Bi_2_Se_3_ NPs were covered with the ITZO thin film. The samples were stored in an ambient environment; therefore, the Bi_2_O_3_ phase was formed on the surface of the Bi_2_Se_3_ NPs. Thus, the orbital signal of Bi 4f. corresponding to the Bi_2_O_3_ lattice was detected. These results indicated that the ITZO covered the surface of Bi_2_Se_3_ NPs.

### Electrical properties

**Figure 8:**
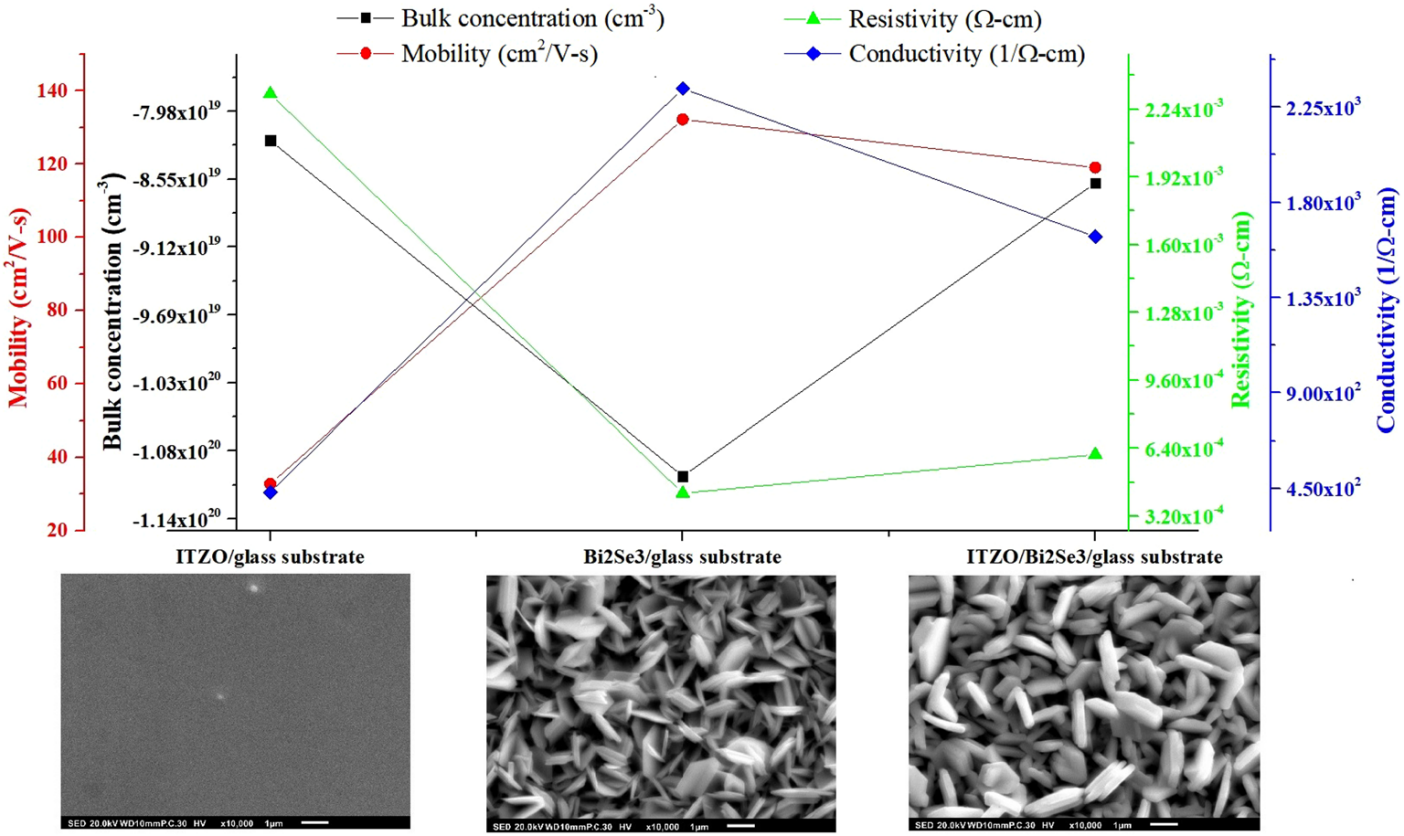
Bulk carrier concentration, resistivity, carrier mobility, and Conductivity of the ITZO thin film, and Bi_2_Se_3_ and ITZO/Bi_2_Se_3_ NPs after annealing at 250 °C.

Figure 8 presents the results for the bulk carrier concentration, resistivity, carrier mobility, and conductivity of the ITZO thin film and Bi_2_Se_3_ and ITZO/Bi_2_Se_3_ NPs after annealing at 250 °C. Table S1 presents the corresponding values, including bulk carrier concentration (− 1 × 10^–19^ cm^−3^), carrier mobility (1 × 10^2^ cm^2^/V-s), resistivity (1 × 10^–4^ Ω-cm), and conductivity (1 × 10^3^ Ω^−1^-cm^−1^), respectively. The bulk carrier concentrations of the ITZO thin film and ITZO/Bi_2_Se_3_ NPs were similar, i.e., approximately − 8 × 10^–19^ cm^−3^, whereas that of the Bi_2_Se_3_ NPs was − 11.04 × 10^–19^ cm^−3^. This suggests that the bulk charged carriers of the Bi_2_Se_3_ NPs had no significant effect on the ITZO/Bi_2_Se_3_ NPs. The carrier mobility ($\mu$) of the ITZO/Bi_2_Se_3_ NPs was 1.2 × 10^2^ cm^2^/V-s, which was 263.6% higher than that of the ITZO thin film. The resistivity (*ρ*) of the ITZO thin film was 23.12 × 10^–4^ Ω-cm, and that of the ITZO/Bi_2_Se_3_ was 6.11 × 10^–4^ Ω-cm, a decrease of 73.57%. For n-type semiconductors, the resistivity (*ρ*) can be described as $\rho =\frac{1}{qn\mu }$, where $q$ represents the electric charge (1.6 × 10^–19^ C), and $n$ represents the carrier concentration, which is similar between ITZO, Bi_2_Se_3_, and ITZO/Bi_2_Se_3_ in this work. Hence, the decrease in resistivity of ITZO/Bi_2_Se_3_ NPs is due to the increase in carrier mobility ($\mu$). The conductivity (σ) of ITZO/Bi_2_Se_3_ NPs was 1.64 × 10^3^ (1/Ω-cm), which was 281.4% higher than that of the ITZO thin film. Because conductivity (σ) is inversely proportional to resistivity (*ρ*), the ITZO/Bi_2_Se_3_ NPs had higher conductivity than the ITZO thin film. The Hall measurement results indicate that the electrical properties of ITZO can be improved through the formation of a heterostructure with Bi_2_Se_3_.

### Proposed mechanism for the enhanced carrier mobility in ITZO/Bi_2_Se_3_ NPs

**Figure 9:**
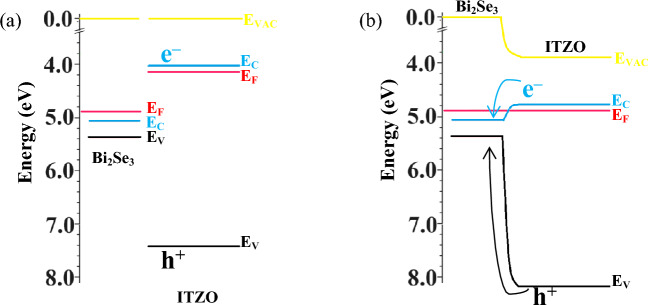
Proposed band diagram of ITZO/Bi_2_Se_3_ NPs (**a**) before and (**b**) after contact.

Bi_2_Se_3_ is identified as an n-type semiconductor with a narrow band-gap of 0.35 eV, and its Fermi level resides within its conduction band^32,57^. On the other hand, ITZO is characterized as an n-type semiconductor with a broad band-gap of 3.40 eV^58^. The band structure at the interface undergoes bending upon the formation of the heterostructure between ITZO and Bi_2_Se_3_. The band diagram of the ITZO/Bi_2_Se_3_ NPs is depicted in Fig. 9, with Fig. 9a illustrating the band diagram before the contact of ITZO and Bi_2_Se_3_ in a thermal equilibrium state. Here, E_VAC_ represents the vacuum level, E_C_ is the conduction band level, E_F_ is the Fermi energy level, and E_V_ is the valence band level. Figure 9b shows the band bending after the contact of ITZO and Bi_2_Se_3_ in a thermal equilibrium state, leading to the migration of electrons (e^‒^) and holes (h^+^) from ITZO to Bi_2_Se_3_. The primary carrier (e^‒^) concentrations progressively increase from the ITZO surface to the interface between ITZO and Bi_2_Se_3_ due to the migration of electrons from ITZO to Bi_2_Se_3_. Additionally, defects such as dislocations and impurities at the ITZO/Bi_2_Se_3_ interface could contribute to decreased carrier concentrations at the ITZO surface of the ITZO/Bi_2_Se_3_ NPs^59^. The carrier mobility, determined through Hall effect measurements, can be estimated using the formula^60^, ${\mu }_{H}=\frac{\sigma }{e{n}_{H}}$, where ${\mu }_{H}$ is the carrier mobility, $e$ is the free electron charge, ${n}_{H}$ is the carrier concentration. Therefore, $\sigma$ and ${n}_{H}$ directly affect the ${\mu }_{H}$. The higher $\sigma$ corresponds to a the larger ${\mu }_{H}$, while a lower ${n}_{H}$ results in a larger ${\mu }_{H}$. As discussed above, the $\sigma$ in ITZO/Bi_2_Se_3_ is significantly higher than that in pure ITZO. Moreover, the extrinsic properties following the contact of ITZO with Bi_2_Se_3_ lead to a decrease in the concentration of primary charged carriers (e^‒^) from the ITZO/Bi_2_Se_3_ interface to the ITZO surface. Simultaneously, the minor charged carriers (h^+^) in ITZO migrate to Bi_2_Se_3_, indicating that e^‒^ in ITZO/Bi_2_Se_3_ NPs exhibits a longer lifetime than that in pure ITZO due to the low recombination rate between e^‒^ and h^+^. Therefore, the synergetic effect after the contact between ITZO and Bi_2_Se_3_ enhances the electrical properties of the ITZO/Bi_2_Se_3_ NPs.

## Conclusions

ITZO/Bi_2_Se_3_ NPs were synthesized on a pure glass substrate through a thermal CVD at 600°C and magnetron sputtering at room-temperature. XRD and HRTEM analyses confirmed the formation of crystalline Bi_2_Se_3_ and disordered ITZO phases. FESEM images indicated that the average thicknesses of the Bi_2_Se_3_ and ITZO/Bi_2_Se_3_ NPs were approximately 79.3 and 239.9 nm, respectively. The Raman spectra indicated that the ITZO coverage suppressed the Se–Se vibration mode of the Bi_2_Se_3_ NPs. XPS measurements revealed the elemental binding energies of the ITZO and Bi_2_Se_3_ lattices, confirming that ITZO covered the Bi_2_Se_3_ NPs, which was consistent with the HRTEM image of the ITZO/Bi_2_Se_3_ NPs. Hall effect measurements of the electrical properties revealed that the bulk carrier concentration of ITZO did not affect through the heterostructure with Bi_2_Se_3_. However, the formation of the heterostructure with Bi_2_Se_3_ increased the carrier mobility and conductivity of ITZO by 263.6% and 281.4%, respectively, and reduced the resistivity of ITZO by 73.57%. These results indicate that the electrical properties of ITZO can be significantly improved through the formation of a heterostructure with Bi_2_Se_3_ owing to its gapless surface state, expanding the potential applications in electronic and thermoelectric devices of ITZO/Bi_2_Se_3_ heterostructures.

## Experimental

### Fabrication of ITZO thin films

The ITZO thin films were deposited on a glass substrate (20 × 20 × 7 mm^3^) via magnetron sputtering under a base pressure of 6.0 × 10^−6^ Torr. The substrate‒target distance was 17 cm. The 3-inch ITZO target was composed of 85 wt% In_2_O_3_, 15 wt% SnO_2_, and 5 wt% ZnO. The deposition was performed at a power of 200 W for 750 s, with the introduction of Ar gas at 25 sccm under a working pressure of 3.0 × 10^−3^ Torr.

### Synthesis of Bi_2_Se_3_ and ITZO/Bi_2_Se_3_ NPs and annealing treatments

Pristine Bi_2_Se_3_ NPs were synthesized on a glass substrate (20 × 20 × 7 mm^3^) via a catalyst-free vapor–solid mechanism using a thermally evaporated deposition process in a horizontal quartz tube furnace. 0.1 g of Bi powder (purity = 99%, 4.78 × 10^−4^ mol, Merck, Darmstadt, Germany) and 0.1 g of Se powder (purity = 99%, 1.27 × 10^−3^ mol, Alfa Aesar, Ward Hill, MA, USA) were used as mixture precursors. They were placed in an alumina boat, which was put in the central heating zone of the quartz tube. The evaporating temperature was 600 °C, the heating rate was 10 °C/min, and the heating was performed at the pressure of 1.0 × 10^−2^ Torr. These conditions were maintained for 20 min to synthesize the Bi_2_Se_3_ NPs. The glass substrate was placed vertically upstream in a quartz tube at 140 °C, 21 cm from the precursor mixture. Pristine Bi_2_Se_3_ NPs were grown on glass substrates. After 60-min, the system was cooled to room-temperature. ITZO thin films were deposited on the Bi_2_Se_3_ NPs for 750 s at a working distance of 17 cm via magnetron sputtering (25 sccm Ar, 200 W) using a 3-inch ITZO target at room-temperature under a working pressure of 3.0 × 10^−3^ Torr. The ITZO thin films, Bi_2_Se_3_ NPs, and ITZO/Bi_2_Se_3_ NPs were placed in the heating zone of a horizontal quartz tube furnace. The furnace was heated to 250 °C at a heating rate of 10 °C/min under 2.0 × 10^−2^ Torr, and then annealing was performed at this temperature for 90 min. Subsequently, the system was cooled down to room-temperature.

### Characterization of ITZO, Bi_2_Se_3_ NPs, and ITZO/Bi_2_Se_3_ NPs

The crystallographic orientations and fine structures of the ITZO thin films and Bi_2_Se_3_ and ITZO/Bi_2_Se_3_ NPs were examined using high-resolution transmission electron microscopy (HRTEM; JEOL JEM-2010) and X-ray diffraction (XRD) spectroscopy (Bruker D2 PHASER, Cu Kα radiation, λ = 1.5405 Å, operating at 40 kV and 30 mA). The sample morphologies were examined using field-emission scanning electron microscopy (FESEM; JOEL JSM-6335F). The binding vibration modes were analyzed via Raman spectroscopy (3D Nanometer-scale Raman PL microspectrometer) using a semiconductor laser with an excitation energy of 2.54 eV. The chemical binding energies and valence states of the elements were analyzed using X-ray photoelectron spectroscopy (XPS; Perkin-Elmer model PHI 1600, operating at 250 W) with Mg Kα X-rays (1253.6 eV). The electrical properties, i.e., the bulk carrier concentration, carrier mobility, resistivity, and conductivity, were evaluated via Hall effect measurements (Ecopia HMS-3000 Hall Measurement System) at room-temperature under an input current of 5 mA and a magnetic flux density of 0535 T.

### Supplementary Information

Supplementary Information.

## Data Availability

The datasets used and/or analysed during the current study available from the corresponding author on reasonable request.

## References

[1] Yang H, Yang W, Su J, Zhang X (2022). Enhancement-mode thin film transistor using amorphous phosphorus-doped Indium-Zinc-Tin-Oxide channel layer. Mater. Sci. Semicond. Process..

[2] Lee HY, Yang IJ, Yoon JH, Jin SH, Kim S, Song PK (2019). Thermoelectric properties of zinc-doped indium tin oxide thin films prepared using the magnetron co-sputtering method. Coatings.

[3] Choi JY, Park IP, Heo SW (2021). Ultra-flexible organic photovoltaics with low-temperature deposited IZTO on a cyclic polymer substrate having excellent mechanical properties. ACS Appl. Mater. Interfaces.

[4] Seo SJ, Choi CG, Hwang YH, Bae BS (2009). High performance solution-processed amorphous zinc tin oxide thin film transistor. J. Phys. D: Appl. Phys..

[5] Jung YS, Seo JY, Lee DW, Jeon DY (2003). Influence of DC magnetron sputtering parameters on the properties of amorphous indium zincoxide thin film. Thin Solid Films.

[6] Kim J, Park J, Yoon G, Khushabu A, Kim JS, Pae S, Cho EC, Yi J (2020). Effect of IGZO thin films fabricated by pulsed-DC and RF sputtering on TFT characteristics. Mater. Sci. Semicond. Process..

[7] Han DS, Kang YJ, Park JH, Jeon HT, Park JW (2014). Influence of molybdenum source/drain electrode contact resistance in amorphous zinc-tin-oxide (a-ZTO) thin film transistors. Mater. Res. Bull..

[8] Rajachidambaram MS, Pandey A, Vilayurganapathy S, Nachimuthu P, Thevuthasan S, Herman GS (2013). Improved stability of amorphous zinc tin oxide thin film transistors using molecular passivation. Appl. Phys. Lett..

[9] Kenji N, Hiromichi O, Akihiro T, Toshio K, Masahiro H, Hideo H (2004). Room-temperature fabrication of transparent flexible thin-film transistors using amorphous oxide semiconductors. Nature.

[10] Lan L, Peng J (2011). High-performance indium-gallium-zinc oxide thin-film transistors based on anodic aluminum oxide. IEEE Trans. Electron. Dev..

[11] Lu YB, Yang TL, Ling ZC, Cong WY, Zhang P, Li YH, Xin YQ (2015). How does the multiple constituent affect the carrier generation and charge transport in multicomponent TCOs of In-Zn-Sn oxide. J. Mater. Chem. C.

[12] Choi P, Lee J, Park H, Baek D, Lee J, Yi J, Kim S, Choi B (2016). Fabrication and characteristics of high mobility InSnZnO thin film transistors. J. Nanosci. Nanotechnol..

[13] Li ZY, Chen SC, Huo QH, Liao MH, Dai MJ, Lin SS, Yang TL, Sun H (2019). Influence of sputtering power on the electrical properties of In-Sn-Zn oxide thin films deposited by high power impulse magnetron sputtering. Coatings.

[14] Zhong W, Li G, Lan L, Li B, Chen R (2018). Effects of annealing temperature on properties of InSnZnO thin film transistors prepared by co-sputtering. RSC Adv..

[15] Sun H, Li ZY, Chen SC, Liao MH, Gong JH, Bai Z, Wang WX (2021). In-Sn-Zn oxide nanocomposited films with enhanced electrical properties deposited by high power impulse magnetron sputtering. Nanomaterials.

[16] Yang H, Yang W, Su J, Zhang X (2022). Enhancement-mode thin film transistor using amorphous phosphorus-doped indium-zinc-tin oxide channel layer. Mater. Sci. Semicond. Process..

[17] Song CW, Kim KH, Yang JW, Kim DH, Choi YJ, Hong CH, Shin JH, Kwon HI, Song SH, Cheong WS (2016). Effects of Mg suppressor layer on the InZnSnO thin-film transistors. J. Semicond. Tech. Sci..

[18] Tomai S, Nishimura M, Itose M, Matuura M, Kasami M, Matsuzaki S, Kawashima H, Utsuno F, Yano K (2012). High-performance thin film transistor with amorphous In_2_O_3_-SnO_2_-ZnO channel layer. Jpn. J. Appl. Phys..

[19] Sheng J, Hong T, Kang D, Yi Y, Lim JH, Park JS (2019). Design of InZnSnO semiconductor alloys synthesized by supercycle atomic layer deposition and their rollable applications. ACS Appl. Mater. Interfaces.

[20] Baek IH, Pyeon JJ, Han SH, Lee GY, Choi BJ, Han JH, Chung TM, Hwang CS, Kim SK (2019). High-performance thin-film transistors of quaternary indium-zinc-tin oxide films grown by atomic layer deposition. ACS Appl. Mater. Interfaces.

[21] Maeng S, Kim H, Choi G, Choi Y, Oh S, Kim J (2020). Investigation of electrical performance and operation stability of RF-sputtered InSnZnO thin film transistors by oxygen-ambient rapid thermal annealing. Semicond. Sci. Technol..

[22] Noviyana I, Lestari AD, Putri M, Won MS, Bae JS, Heo YW, Lee HY (2017). High mobility thin film transistors based on amorphous indium zinc tin oxide. Materials.

[23] Nam Y, Yang JH, Jeong P, Kwon OS, Pi JE, Cho SH, Hwang CS, Ahn J, Ji S, Park SHK (2017). Effect of a rapid thermal annealing process on the electrical properties of an aluminum-doped indium zinc tin oxide thin film transistor. Phys. Status Solidi.

[24] Yang JW, Na YB, Shin JH, Hong CH, Seo WH, Kim KH, Song CW, Song SH, Kwon HI, Cheong WS (2017). Effects of Er-doping on amorphous InZnSnO/InZnSnO: Er double-channel thin-film transistors. J. Nanosci. Nanotechnol..

[25] Su J, Yang H, Yang W, Zhang X (2022). Electrical characteristics of tungsten-doped InZnSnO thin film transistors by RF magnetron sputtering. J. Vac. Sci. Technol. B.

[26] Zhang H, Liang L, Wang X, Wu Z, Cao H (2022). Praseodymium-doped In-Sn-Zn-O TFTs with effective improvement of negative-bias illumination stress stability. IEEE Trans. Electron. Devices.

[27] Choi KH, Kim JY, Lee YS, Kim HJ (1999). ITO/Ag/ITO multilayer films for the application of a very low resistance transparent electrode. Thin Solid Films.

[28] Miao D, Jiang S, Shang S, Chen Z (2014). Infrared reflective properties of AZO/Ag/AZO trilayers prepared by RF magnetron sputtering. Ceram. Int..

[29] Lee SM, Koo HW, Kim TW, Kim HK (2018). Asymmetric ITO/Ag/ZTO and ZTO/Ag/ITO anodes prepared by roll-to-roll sputtering for flexible organic light-emitting diodes. Surf. Coat. Technol..

[30] Song S, Yang T, Xin Y, Jiang L, Li Y, Pang Z, Lv M, Han S (2010). Effect of GZO thickness and annealing temperature on the structural, electrical and optical properties of GZO/Ag/GZO sandwich films. Curr. Appl. Phys..

[31] Jeong JA, Park YS, Kim HK (2010). Comparison of electrical, optical, structural, and interface properties of IZO-Ag-IZO and IZO-Au-IZO multilayer electrodes for organic photovoltaics. J. Appl. Phys..

[32] Wang CC, Chang YS, Lin PT, Shieu FS, Shih HC (2022). Fabrication, characterization and optical properties of Au-decorated Bi2Se3 nanoplatelets. Sci. Rep..

[33] Zhang H, Liu CX, Qi XL, Dai X, Fang Z, Zhang SC (2009). Topological insulators in Bi_2_Se_3_, Bi_2_Te_3_ and Sb_2_Te_3_ with a single Dirac cone on the surface. Nat. Phys..

[34] Wang CC, Shieu FS, Shih HC (2021). Photosensing and characterizing of the pristine and In-, Sn-doped Bi_2_Se_3_ nanoplatelets fabricated by thermal V-S process. Nanomaterials.

[35] Irfan B, Sahoo S, Gaur APS, Ahmadi M, Guinel MJF, Katiyar RS, Chatterjee R (2014). Temperature dependent Raman scattering studies of three dimensional topological insulators Bi_2_Se_3_. J. Appl. Phys..

[36] Schönherr P, Collins-McIntyre LJ, Zhang S, Kusch P, Reich S, Giles T, Daisenberger D, Prabhakaran D, Hesjedal T (2014). Vapour-liquid-solid growth of ternary Bi_2_Se_2_Te nanowires. Nanoscale Res. Lett..

[37] Meyer N, Geishendorf K, Walowski J, Thomas A, Munzenberg M (2020). Photocurrent measurements in topological insulator Bi_2_Se_3_ nanowires. Appl. Phys. Lett..

[38] Yue C, Jiang S, Zhu H, Chen L, Sun Q, Zhang DW (2018). Device applications of synthetic topological insulator nanostructures. Electronics.

[39] Tian W, Yu W, Shi J, Wang Y (2017). The property, preparation and application of topological insulators: A review. Materials.

[40] Fei F, Zhang S, Zhang M, Shah SA, Song F, Wang X, Wang B (2020). The material efforts for quantized Hall devices based on topological insulators. Adv. Mater..

[41] Hsieh D, Xia Y, Wray L, Qian D, Pal A, Dil JH, Meier F, Osterwalder J, Bihlmayer G, Kane CL, Hor YS, Cava RJ, Hasan MZ (2009). First direct observation of spin-textures in topological insulators: Spin-resolved ARPES as a probe of topological quantum spin Hall effect and Berry’s phase. Science.

[42] Carreras P, Antony A, Rojas F, Bertomeu J (2011). Electrical and optical properties of Zn–In–Sn–O transparent conducting thin films. Thin Solid Films.

[43] Sun H, Li ZY, Chen SC, Liao MH, Gong JH, Bai Z, Wang WX (2021). In-Sn-Zn oxide nanocomposite films with enhanced electrical properties deposited by high-power impulse magnetron sputtering. Nanomaterials.

[44] Wool, R. P., & Sun, X. S. Bio-based polymers and composites. Academic Press, pp 483‒522 (2005).

[45] Xu K, Li Y, Xiong OuJ, Su X (2018). Activated amorphous carbon with high-porosity derived from camellia pollen grains as anode materials for lithium/sodium ion batteries. Front. Chem..

[46] Zhang J, Peng Z, Son A, Zhao Y, Xiong Y, Peng B, Wang J, Dresselhaus MS, Xiong Q (2011). Raman spectroscopy of few-quintuple layer topological insulator Bi_2_Se_3_ nanoplatelets. Nano Lett..

[47] Wang CC, Lin PT, Shieu FS, Shih HC (2021). Enhanced photocurrent of the Ag interfaced topological insulator Bi_2_Se_3_ under UV- and visible-light radiations. Nanomaterials.

[48] Ahmed R, Lin Q, Xu Y, Zangari G (2019). Growth, morphology and crystal structure of electrodeposited Bi_2_Se_3_ films: Influence of the substrate. Electrochim. Acta.

[49] Jurczyszyn M, Sikora M, Chrobak M, Jurczyszyn L (2020). Studies of surface states in Bi_2_Se_3_ induced by the Bi_Se_ substitution in the crystal subsurface structure. Appl. Surf. Sci..

[50] Ahmed R, Xu Y, Sales MG, Lin Q, McDonnell S, Zangari G (2018). Synthesis and material properties of Bi_2_Se_3_ nanostructures deposited by SILAR. J. Phys. Chem. C.

[51] Shinde DV, Ahn DY, Jadhav VA, Lee DY, Shrestha NK, Lee JK, Lee HY, Mane RS, Han SH (2014). A coordination chemistry approach for shape controlled synthesis of indium oxide nanostructures and their photoelectrochemical properties. Mater. Chem. A.

[52] Wang L, Li J, Wang Y, Yu K, Tang X, Zhang Y, Wang S, Wei C (2016). Construction of 1D SnO_2_-coated ZnO nanowire heterojunction for their improved n-butylamine sensing performances. Sci. Rep..

[53] Wagner CD, Riggs WM, Davis LE, Moulder JF, Muilenberg GE (1979). Handbook of x-ray photoelectron spectroscopy.

[54] Wang CC, Lin WC, Shieu FS, Shih HC (2019). Enhanced optoelectronic properties of thermally evaporated Sb-doped ZnO nanowires via defect structures. AIP Adv..

[55] Acharyya D, Saini A, Bhattacharyya P (2018). Influence of rGO cladding in improving the sensitivity and selectivity of ZnO nanoflowers-based alcohol sensor. IEEE Sens. J..

[56] Zhang G, Qin H, Teng J, Guo J, Guo Q, Dai X, Fang Z, Wu K (2009). Quintuple-layer epitaxy of thin films of topological insulator Bi_2_Se_3_. Appl. Phys. Lett..

[57] Suh J, Fu D, Liu X, Furdyna JK, Yu KM, Walukiewicz W, Wu J (2014). Fermi-level stabilization in the topological insulators Bi_2_Se_3_ and Bi_2_Te_3_: origin of the surface electron gas. Phys. Rev. B.

[58] Li Y, Zhao C, Zhu D, Cao P, Han S, Lu Y, Fang M, Liu W, Xu W (2020). Recent advances of solution-processed heterojunction oxide thin-film transistors. Nanomaterials.

[59] Linhart WM, Veal TD, King PDC, Koblmüller G, Gallinat CS, Speck JS, McConville CF (2010). Surface, bulk, and interface electronic properties of nonpolar InN. Appl. Phys. Lett..

[60] Wang S, Li H, Lu R, Zheng G, Tang X (2013). Metal nanoparticle decorated n-type Bi_2_Te_3_-based materials with enhanced thermoelectric performances. Nanotechnology.

