# Supplementary material for: Enhanced electrical properties of amorphous In-Sn-Zn oxides through heterostructuring with Bi2Se3 topological insulators
Source: Sci Rep. 2024 Jan 2;14:195. doi: 10.1038/s41598-023-50809-7 (PMC10762253; doi:10.1038/s41598-023-50809-7)
Supplement: Supplementary file 1 — Supplementary Information. [file 41598_2023_50809_MOESM1_ESM.docx]

| 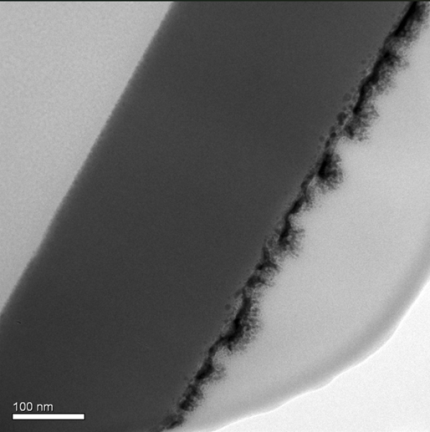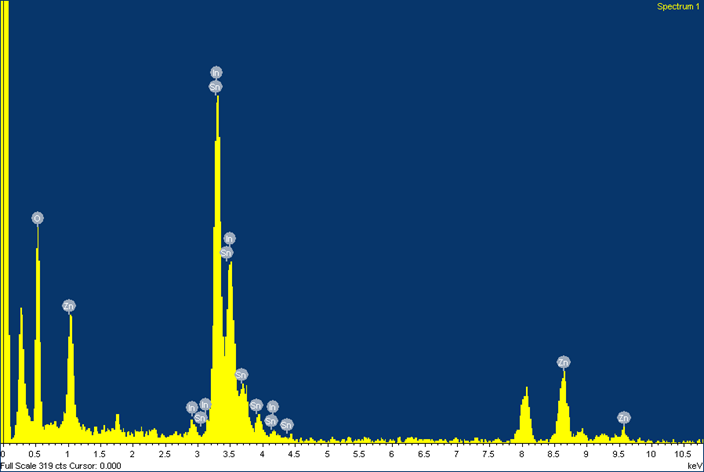  ITZO  Glass  (a) |
| --- |
| 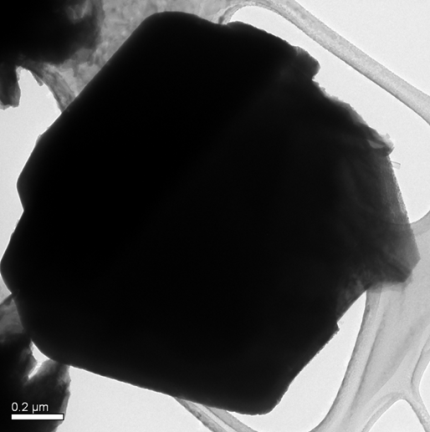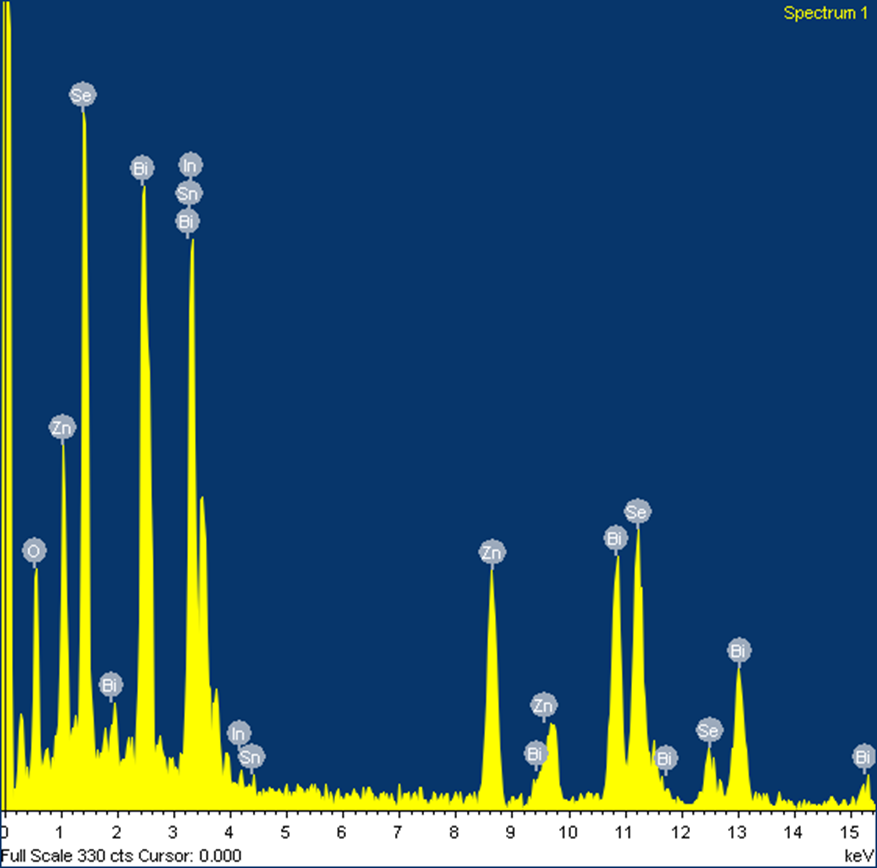  ITZO/Bi_2_Se_3_  (b) |
| **Figure S1**. TEM-EDS of (a) ITZO thin film and (b) ITZO/Bi_2_Se_3_ NP after annealing at 250 ℃. |

| 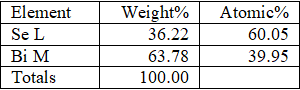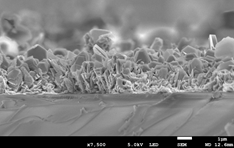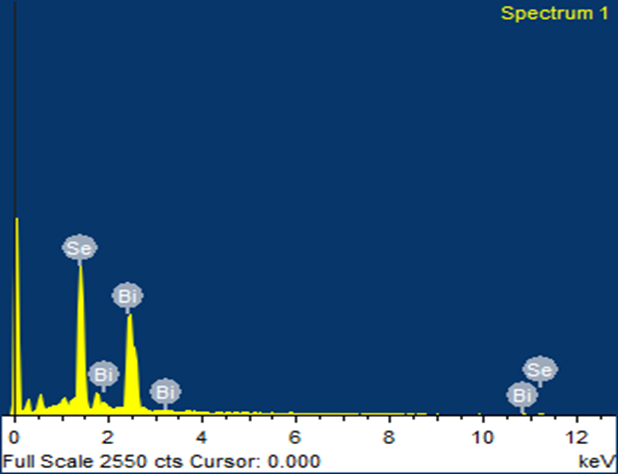  Bi_2_Se_3_  Glass substrate  (a) | 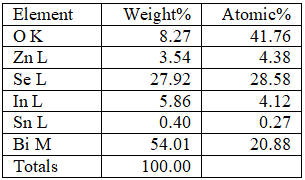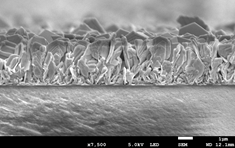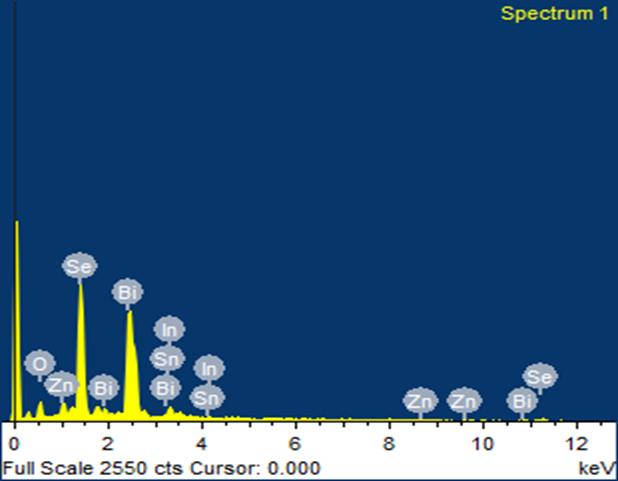  ITZO/Bi_2_Se_3_  Glass substrate  (b) |
| --- | --- |
| **Figure S2**. SEM-EDS of (a) Bi_2_Se_3_ and (b) ITZO/Bi_2_Se_3_ NPs before annealing at 250℃. | |

(b)

(a)

| 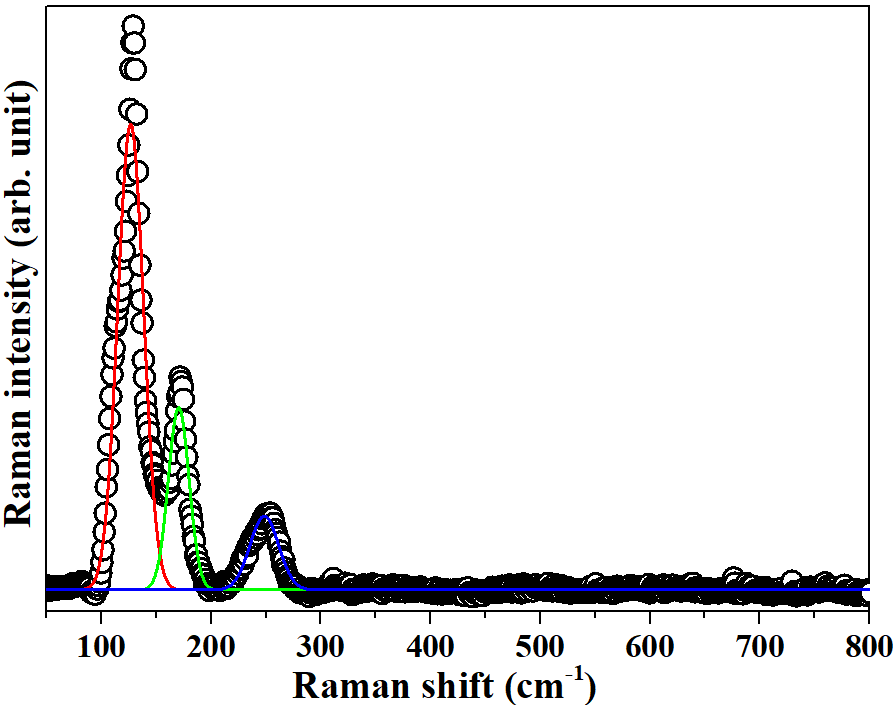  **126.69**  **170.51**  248.49  (c)  (d) | 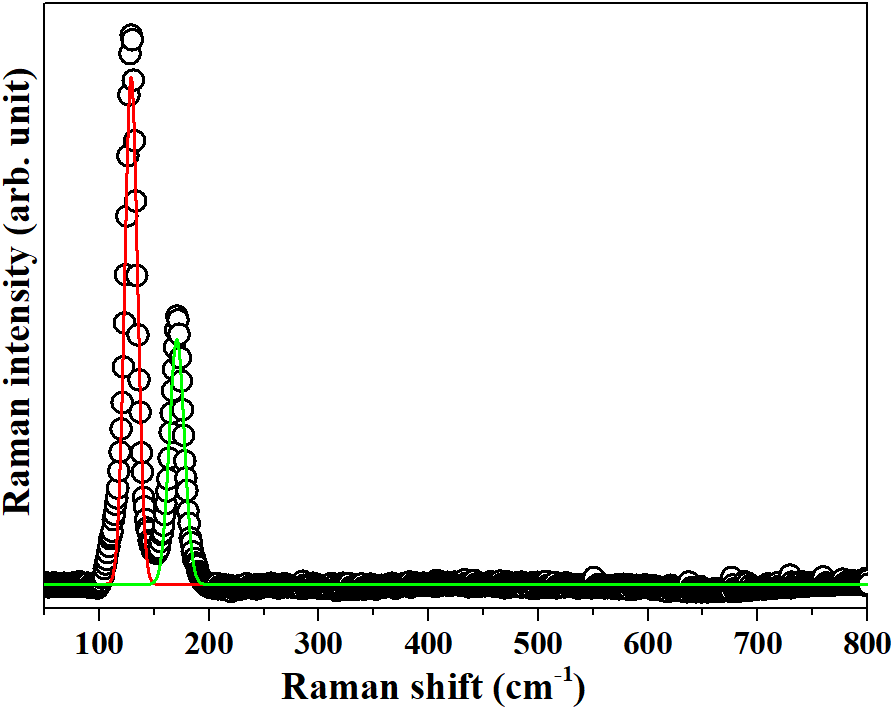  **128.99**  **170.64** |
| --- | --- |
| 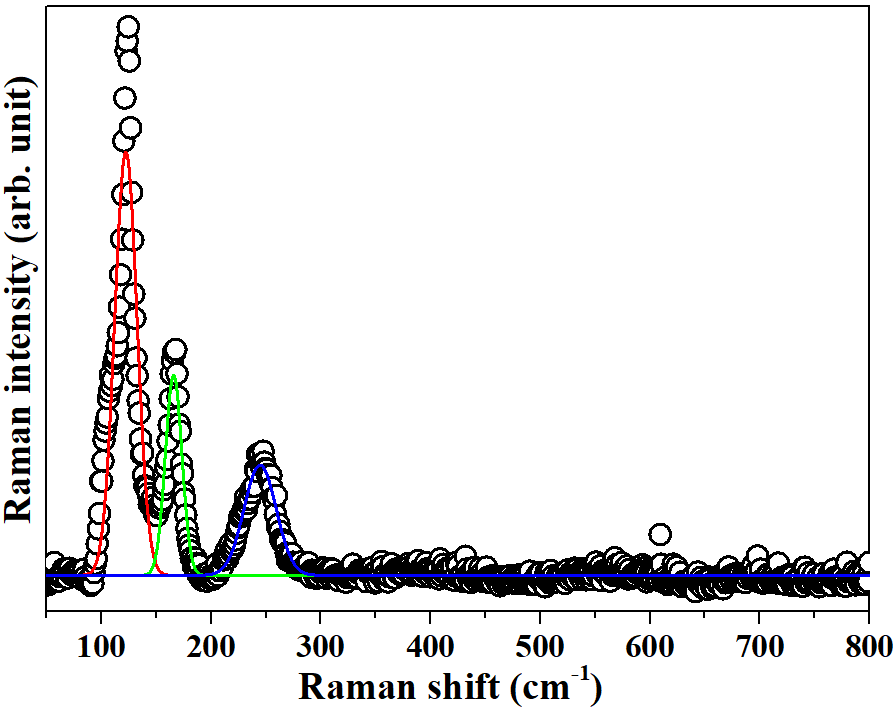  **122.37**  **165.90**  244.67 | 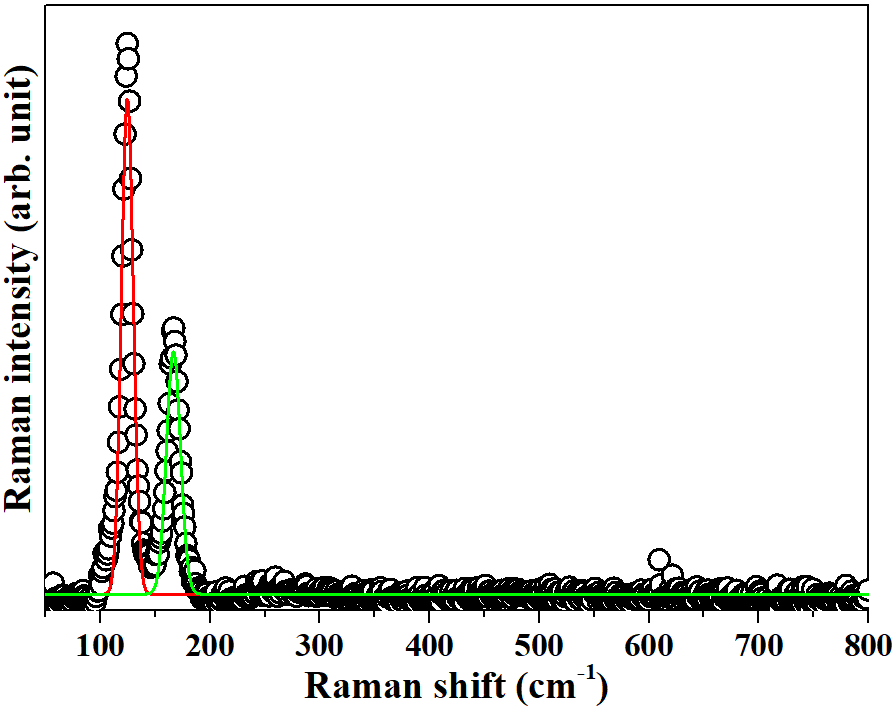  **124.66**  **166.67** |
| **Figure S3**. Raman spectra of Bi_2_Se_3_ and ITZO/Bi_2_Se_3_ NPs (a) and (b) before, and (c) and (d) after annealing at 250 ℃. | |

| **Table S1**. Relevant values of the bulk carrier concentration, carrier mobility, resistivity, and conductivity of the ITZO thin film, and Bi_2_Se_3_ and ITZO/Bi_2_Se_3_ NPs after annealing at 250 ℃. | | | |
| --- | --- | --- | --- |
| **Samples** | **ITZO** | **Bi_2_Se_3_** | **ITZO/Bi_2_Se_3_** |
| **Bulk carrier concentration (-1🞨10^-19^ cm^-3^)** | 8.22 | 11.04 | 8.58 |
| **Carrier mobility (1🞨10^2^ cm^2^/V-s)** | 0.33 | 1.32 | 1.20 |
| **Resistivity (1🞨10^-4^ Ω-cm)** | 23.12 | 4.27 | 6.11 |
| **Conductivity (1🞨10^3^ Ω^-1^-cm^-1^)** | 0.43 | 2.34 | 1.64 |
